# Clinical characteristics, biomarkers and prevalence of cerebral amyloid angiopathy in Alzheimer's disease: Applying the Boston criteria v2.0 in a memory clinic population

**DOI:** 10.1002/alz70856_096510

**Published:** 2025-12-24

**Authors:** Hadrien M Lalive, Federica Ribaldi, Augusto J. Mendes, Chen Wang, Christian Chicherio, Max Scheffler, Karl‐Olof Lovblad, Giovanni B Frisoni, Aurelien Lathuiliere

**Affiliations:** ^1^ Geneva Memory Center, Geneva University Hospitals and University of Geneva, Geneva, Switzerland; ^2^ Laboratory of Neuroimaging of Aging (LANVIE), University of Geneva, Geneva, Switzerland; ^3^ Center for Interdisciplinary Study of Gerontology and Vulnerability (CIGEV), University of Geneva, Geneva, Switzerland; ^4^ Division of Radiology, Geneva University Hospitals, Geneva, Switzerland; ^5^ Division of Neuroradiology, Geneva University Hospitals, Geneva, Switzerland

## Abstract

**Background:**

Diagnosing cerebral amyloid angiopathy (CAA) is essential when determining Alzheimer's disease (AD) patients’ eligibility for anti‐amyloid immunotherapy since CAA is associated with amyloid‐related imaging abnormalities. The Boston criteria are widely used in memory clinics to identify older adults with a high probability of CAA; however, the prevalence and clinical characteristics of CAA among cognitively impaired patients with AD remain unclear. This retrospective study aimed to determine the prevalence of CAA in cognitively impaired older adults with biomarker‐confirmed AD (CI‐AD) using the updated Boston criteria (v2.0), and to compare the cognitive, clinical, and biomarker profiles of CI‐AD patients with and without CAA.

**Method:**

415 patients (mean age 73.8 ± 7.0 years) with probable AD, confirmed by lumbar puncture or positron emission tomography (PET) biomarkers, were retrospectively identified from the Geneva University Hospital Memory Center's database (June 2012 ‐ July 2024). MRI scans were analyzed by a board‐certified radiologist or neuroradiologist, independently reviewed by a trained image analyst, and imaging diagnoses confirmed by an experienced neurologist, the latter two blinded to clinical data. Participants were classified as having a high (AD‐CAA) or low probability of CAA (AD‐nCAA) using the Boston criteria. Patient characteristics and biomarker distributions were compared between groups using the Chi‐squared test or Fisher's exact test for categorical variables, and the Mann‐Whitney U test for continuous variables.

**Result:**

29% (*n* = 119) of AD patients were classified as AD‐CAA, whereas 71% (*n* = 296) were classified as AD‐nCAA. Clinical severity, global cognition, verbal episodic memory, and executive functions were comparable between groups. AD‐CAA patients were older, more likely to use antiplatelet therapy, and exhibited a higher prevalence of cardiovascular disease, despite having similar cardiovascular risk factors. No significant differences between groups were observed in fluid AD biomarkers, PET amyloid burden, or medial temporal atrophy assessed through imaging.

**Conclusion:**

The prevalence of CAA among AD patients was lower than pathology‐based estimates. AD patients from memory clinics may exhibit comparable cognitive profiles and similar patterns of AD biomarkers regardless of their CAA probability. These findings underscore the importance of further research in the application of the Boston criteria in memory clinic patients with AD.

